# The Differential Consequences of Fear, Anger, and Depression in Response to COVID-19 in South Korea

**DOI:** 10.3390/ijerph19116723

**Published:** 2022-05-31

**Authors:** Jounghwa Choi, Kyung-Hee Kim

**Affiliations:** 1 Department of Advertising and Public Relations, Hallym University, Chuncheon-si 24252, Korea; 2 The Media School, Hallym University, Chuncheon-si 24252, Korea; khkim@hallym.ac.kr

**Keywords:** COVID-19, fear, anger, depression, social distancing, stigmatization

## Abstract

Studies on previous outbreaks of contagious diseases suggest that the impact of the emotions associated with an epidemic can be greater than that of the epidemic in terms of the number of people affected. This study explores the relationships between the three most commonly expressed emotional responses to the COVID-19 pandemic (fear, anger, and depression) and two outcome variables (compliance with the social-distancing policy and the stigmatization of those infected by COVID-19). A large online, public opinion survey was conducted in South Korea (*n* = 1000) between 4 and 11 June 2020, which was between the first and the second waves of COVID-19. A series of regression analyses suggest that the emotional response was accompanied by differential behavioral and perceptual consequences. Fear was consistently positively related to all indicators of compliance with social-distancing policies (the voluntary practice of social distancing, support for the “routine-life-distancing” policy, and support for stronger social-distancing policies). Anger was positively related to both stigmatization indicators (responsibility attribution and stigmatizing attitude toward people infected with COVID-19). Finally, depression showed negative relationships with support for the “routine-life-distancing” policy and for stronger social-distancing policies but a positive relationship with the voluntary practice of social distancing. By examining whether and how certain types of emotional responses are more or less related to compliance with social distancing and stigmatization, the present study provides practical implications for effective public communication during an epidemic such as COVID-19.

## 1. Introduction

The immediate responses among people to a pandemic tend to be emotional, and such emotions can lead to significant consequences. Indeed, in the middle of COVID-19, we frequently encounter emotional responses: frightened people engage in panic buying; angry people yell at and even attack those they believe caused COVID-19; many people say that they are depressed due to limited freedom and social interactions and economic hardship. These three types of emotional responses—fear, anger, and depression—might be the most commonly expressed and observed responses during the recent COVID-19 outbreak.

Taylor has argued that failing to address people’s emotions could complicate health authorities’ epidemic control efforts [1]. Risk communication scholars have addressed how emotions play an important role in individuals’ behaviors in situations involving risk. Emotions play a critical role in risk appraisal and can influence information-processing and decision-making regarding a risk [2,3]. The appraisal tendency framework (ATF) [4], in particular, suggests that each discrete emotion has distinct cognitive and motivational properties and thus involves a unique appraisal tendency and predicts different cognitive and behavioral outcomes [5]. Therefore, it is necessary to understand how each emotional response to COVID-19 is associated with differential consequences, and such an understanding can offer important insights into pandemic management [1].

At least two kinds of behavioral and perceptual consequences require special attention to manage a pandemic such as COVID-19: compliance with the social-distancing policy and the stigmatization of infected individuals. Exploring these two outcomes is critical: social distancing is an essential instrument for epidemic control and can be implemented through public cooperation and compliance [1]; the stigma associated with infectious diseases can prevent people from accessing health authorities and thus become a major barrier during a pandemic emergency [6].

Therefore, this study aims to explore the relationships between the three most commonly expressed emotional responses in the COVID-19 pandemic (fear, anger, and depression) and two behavioral and perceptual responses (compliance with the social-distancing policy and the stigmatization of individuals infected by COVID-19). By examining whether and how certain types of emotional responses are more or less related to a particular behavioral response, the present study aims to provide practical implications for effective public communication during an epidemic. The following section discusses how fear, anger and depression can be related to compliance with social-distancing policies and the stigmatization of people infected with COVID-19.

### 1.1. Fear

Fear is the most studied emotion and has been a central component in health and risk communication. According to the ATF, fear arises from appraisals of uncertainty about what is happening and a lack of situational control over negative events [5]. Therefore, fear is associated with the motivation to escape and accompanies avoidance behaviors in response to threats or action tendencies to reduce uncertainty [7,8,9]. Although theories such as the extended parallel process model [10] suggest that fear often functions as an inhibitor of adaptive behaviors when perceptions exist of a lack of efficacy, fear has been considered an important motivator of healthy or risk prevention behaviors. In Tannenbaum et al.’s meta-analysis, fear appeal messages were shown to be consistently effective for influencing healthy attitudes, intentions, and behaviors; the effects were not constrained by certain conditions [11]. A number of health and risk communication studies have shown that fear is related to an active response to a threat or risk [12,13].

Studies conducted in the context of an epidemic have also revealed consistent results. A study conducted in the context of the 2015 Middle East Respiratory Syndrome coronavirus (MERS-CoV) outbreak in South Korea reported a positive relationship between fear and preventive behavior [14]. More recent studies in the COVID-19 context have also shown that fear is positively related to engagement in preventive behaviors [15,16].

Since empirical evidence suggests that fear is positively related to an active response to a threat, fear regarding COVID-19 is expected to lead to greater participation and support for governments’ social-distancing policies. In particular, because fearful people perceive greater risk and less control over their situations [12,17], they may be more compliant with government policies. Given this rationale, the following hypothesis is proposed:

**Hypothesis** **1.** *Fear will be positively related to compliance with the government’s social-distancing policy in response to COVID-19*.

### 1.2. Anger

Anger is the most commonly experienced response to major negative events, such as terrorist attacks, financial crises, and environmental health crises [12,18,19]. Anger is also frequently observed in outbreaks of contagious diseases such as Ebola and MERS-CoV [20,21]. While anger is often experienced together with fear, the extant literature suggests that they are very different in nature. According to the ATF, anger involves appraisals of certainty about what happened and a sense of situational control over negative events [5]. Due to this appraisal tendency, angry people tend to perceive lower risk [12,22] and tend to engage more in risk-seeking behaviors, whereas fearful people tend to be more risk averse [17]. In addition, anger tends to be related to the belief that other individuals are responsible for a negative event [23], which explains why angry people show a stronger desire to change the situation, particularly by taking actions against other people or obstacles [24]. Several studies have reported that anger can serve as an important motivator for activist behaviors.

These findings suggest that anger may lead people to be less supportive of the government’s course of action to control COVID-19, as angry people have a greater sense of control and are less likely to perceive risk. In fact, recent studies in the COVID-19 context have found that anger is negatively related to the perceived importance of government restriction, self-reported compliant behavior [25] or support for government responses to COVID-19 [26]. The findings from Han et al. could be interpreted in this sense: in a survey of South Korean adults in the COVID-19 context, angry people were more likely to consider false claims to be scientifically credible [27]. This finding may indicate that angry people are less likely to rely on public health authorities. Therefore, the following hypothesis is proposed.

**Hypothesis** **2.** *Anger will be negatively related to compliance with the government’s social-distancing policy in response to COVID-19*.

In addition, anger is expected to be related to the stigmatization of individuals infected with COVID-19. Anger involves the appraisal of a negative event as having high controllability [5]. When control over negative events is perceived to be high, people are more likely to make personal attributions over situational attributions [28,29]. Therefore, angry people tend to attribute the responsibility for negative events to other individuals, thus exhibiting behavior that is more aggressive toward those who bear the negative condition or those who are deemed to have caused the negative situation [30]. A study reported that anger leads to behavior that is more aggressive more so than do emotions such as fear or regret [30]. Consistent with previous findings, Zhang et al. found that anger is positively related to the public stigma related to COVID-19 among Chinese people [31]. These findings suggest that experiencing anger in the context of COVID-19 will lead to greater personal attribution to those diagnosed with COVID-19 and a greater stigmatizing attitude toward them. Therefore, the following hypothesis is proposed.

**Hypothesis** **3.** *Those with higher anger will engage in greater stigmatization toward individuals infected with COVID-19*.

### 1.3. Depression

Negative events, such as a pandemic or natural disaster, can cause society-wide mental health problems. Several studies have already reported a deterioration in mental health in the population due to COVID-19 [32,33] and a sharp increase in depression, which is an important indicator of mental health [34,35].

Depression as an emotional or psycho-affective disorder involves “the loss of interest or pleasure in everyday activities, low energy, and negative thoughts about the self, about life, and about the future” [36]. Sadness and despair are considered the central emotions involving depression [36]. As sadness concerns the cognitive tendency to underestimate the controllability of a situation and to evaluate self-esteem as low [37], depression tends to be related to lower motivations for behavioral change. Consistently, studies have found that depression is related to the attribution of negative life events to uncontrollable causes [38], which suggests that depressive individuals perceive a low sense of control over negative situations. Research findings on the hopelessness theory of depression [39] suggest that hopeless depression affects individuals’ motivation such that it retards the initiation of voluntary responses, particularly when the person’s expectation of positive change is low.

Empirical studies have shown that people with higher (vs. lower) depressive symptoms are more likely to use emotion- rather than task-oriented coping [40]. Similarly, individuals’ current depression was positively related to ineffective escapism [41]. A recent study conducted in South Korea in the COVID-19 context has also provided evidence consistent with these previous findings. Park, Lee, Sul and Chung found that as people experience less optimism about the COVID-19 situation, they become more likely to report greater depressive symptoms, exhibiting lower perceptions of the importance of social distancing and less voluntary motivation to engage in preventive behavior [42]. Given this rationale, the following hypothesis is proposed:

**Hypothesis** **4.** *Depression will be negatively related to compliance with the government’s social-distancing policy*.

### 1.4. Research Context: Early Stage of COVID-19 in South Korea

South Korea experienced the first wave of the COVID-19 pandemic in February and March 2020. After that, the number of confirmed cases was maintained at the 2-digit level, leading the government to relax its social-distancing policies sequentially: from “intensified social distancing” to less strict social distancing on 19 April 2020 and to so-called “routine-life distancing” on 6 May 2020. However, immediately after the second step, sporadic small group infections began to arise, and the situation progressed into the second wave in August 2020.

As of June 2020, although the country’s efforts have generally been hailed as a model of COVID-19 containment [43], the emotional expression and responses discussed above have also been common among Koreans. The lack of face masks has been decried in fear of the virus, an angry public petitioned for the impeachment of President Moon, and the number of cases involving self-harm surged, suggesting that the pandemic is aggravating psychological distress [44,45,46]. Stigmatization toward certain groups of individuals was also observed. For example, Shincheonji is a fringe religious group that was blamed as a key player in the first wave. People who visited Itaewon, a nightlife district in Seoul, were also blamed because cluster infections were reported in May 2020 linked to this area, which scared the public.

Early-stage pandemic control is crucial because people can easily experience psychological distress due to a high level of uncertainty [1]. By examining the aforementioned hypotheses in the context of the early stage of the COVID-19 pandemic in South Korea, this study expects to provide implications for initial response strategies for pandemic control.

## 2. Methods

### 2.1. Data Collection

This study utilized data from a large online public opinion survey concerning COVID-19 in South Korea conducted between 4 and 11 June 2020. The online survey investigated perceptions and behaviors related to COVID-19 and included relevant psychological variables. The data for this study were collected by a research firm utilizing the largest online survey panel in Korea. Quota sampling was utilized proportionate to gender, age and residential area. To ensure the quality of the responses, the participants were screened out based upon their qualifications, response times, and survey completion. This study was approved by the institutional review board at the institution with which the authors are affiliated, and informed consent was obtained from all the participants. In total, 1000 participants aged 19 to 88 years completed the survey; the females numbered 506 (50.6%), and the males numbered 494 (49.4%) (see Table 1 for the demographic characteristics of the participants).

### 2.2. Survey Instrument and Measures

Before the main survey, a pilot study was conducted with college students (*n* = 19). The participants in the pilot study completed the survey and provided their comments on the questionnaires via two focus group sessions on Zoom. The questionnaires were finalized based on comments from the pilot study.

*Emotions.* The participants were asked to indicate the extent to which they felt each emotional state since the occurrence of the COVID-19 outbreak on a five-point Likert-type scale (1 = “never” to 5 = “always”). Fear was assessed with four items used by Chadwick [47]: “fearful,” “worried,” “afraid,” and “anxious.” Anger was assessed with four items (“angry,” “annoyed,” “frustrated,” and “mad”) that are commonly employed in other studies [48,49,50]. Depression was measured with the PROMIS (Patient-Reported Outcomes Measurement Information System) four-item depression scale (“hopeless,” “worthless,” “helpless,” and “depressed”) [51], as the measure is brief but reasonably efficient [52]. The questionnaires were counterbalanced by emotion type.

*Compliance with social-distancing policies.* Compliance with social-distancing policies was assessed in terms of (1) the voluntary practice of social distancing, (2) support for the “routine-life-distancing” policy, and (3) support for stronger social-distancing policies. The voluntary practice of social distancing was assessed by asking the participants how often they had engaged in the following behaviors since the announcement of the “routine-life-distancing” policy on 6 May 2020: “refraining from participating in private meetings or events or visiting crowded places,” “keeping a distance of two arm lengths when having a private or official meeting,” and “practicing self-quarantine for a while after visiting crowded places” (1 = “never” to 5 = “always”). Support for the “routine-life-distancing” policy was measured with three items: “I support the policy,” “I will actively comply with the rules,” and “I will actively cooperate with the policy” (1 = “strongly disagree” to 5 = “strongly agree”). Finally, the participants were asked to indicate on three five-point bipolar items what they would do if a stronger social-distancing policy was imposed again due to an increase in community infections: 1 = “will not cooperate,” 5 = “will actively cooperate”, 1 = “will not support the policy,” 5 = “will support the policy”, 1 = “will not care about the rules,” or 5 = “will actively abide by the rules.” The items for each variable were averaged, and these three variables concerning social distancing were used as the dependent variables in the analysis.

*Stigmatization.* Stigmatization of those who became infected was measured in terms of responsibility attribution and attitude toward those who became infected with COVID-19. Responsibility attribution was measured by asking about the extent to which the following subjects were responsible for the current COVID-19 situation (in reference to Chang, Kim, Shim, & Ma, 2016; Choi & Jeong, 2020) (1 = “not at all responsible,” 5 = “responsible very much.”): (1) each individual who became infected with COVID-19, (2) the followers of Shincheonji, and (3) visitors to crowded facilities such as Itaewon. To assess attitudes toward those who became infected with COVID-19, the participants were asked the extent to which they agreed with the following statements, devised by the authors (1 = “strongly disagree” to 5 = “strongly agree”): “people infected with COVID-19 were ‘reckless,’ ‘irresponsible,’ and ‘lacked consideration for others.’” The items for attribution and attitude were averaged to create a composite scale.

*Control variables.* The demographic variables included in the analysis as control variables were gender (male = 0, female = 1), age, educational level, and household income. In addition, several variables were included relevant to specific COVID-19 circumstances. Participant residence was considered by a dichotomized variable (1 = Daegu and Gyungbuk area, 2 = others) because the Daegu and Gyungbuk areas were heavily affected in the first wave. A variable named “confirmed case” was coded as 1 if a participant or one of their acquaintances had become infected with COVID-19 or had been required to self-quarantine, while the others were coded as 0. This variable was considered to control for the influence of personal involvement with COVID-19. Since mass or small cluster infections occurred among religious groups, especially among those who attended the Christian church Shincheonji, these religious groups received public criticism. Therefore, religion was included, where Shincheonji followers or Christians were coded as 1 and others as 0. Support for Mr. Moon, the President of South Korea, was also included because compliance with government policy can be influenced by one’s political position. Finally, perceptions of severity and susceptibility were included because previous studies have suggested that they have a strong influence on health- and risk-related attitudes, intentions, and behaviors.

Descriptive statistics of the key variables are presented in Table 2.

## 3. Analysis and Results

To examine the hypothesized relationships, hierarchical regression analyses were conducted to test the independent relationship of each discrete emotion with the dependent variables, considering the other control variables. The demographic variables were entered in the first block, and the other control variables were entered in the second block. Finally, the three emotions were entered into the third block. No multicollinearity issue was found (all *VIFs* < 2.0). For a few variables, skewness and kurtosis statistics greater than 1 were detected, but an analysis with transformed scores revealed results similar to those with original scores. Therefore, the results with the original scores are reported.

The first hypothesis concerns the positive relationship between fear and compliance with the government’s social-distancing policy in response to COVID-19. Consistent with the prediction, fear showed a positive association with all three indicators of policy compliance: *β* = 0.095 [95% CI = 0.011 to 0.179], *p* = 0.026 for the voluntary practice of social distancing; *β* = 0.114 [95% CI = 0.038 to 0.189], *p* = 0.003 for support for the “routine-life-distancing” policy; and *β* = 0.088 [95% CI = 0.010 to 0.167], *p* = 0.027 for support for stronger social-distancing policies. Therefore, H1 was supported.

The second hypothesis suggests a negative relationship between anger and compliance with the government’s social-distancing policy. The expected relationship was found only for the voluntary practice of social distancing: *β* = −0.083 [95% CI = −0.162 to −0.004], *p* = 0.04. The relationships between anger and support for the “routine-life-distancing” policy (*β* = 0.027 [95% CI = −0.044 to 0.099], *p* = 0.454) and that for stronger social-distancing policies (*β* = 0.053 [95% CI = −0.021 to 0.127], *p* = 0.158) were not significant. Therefore, the data supporting H2 are limited.

The third hypothesis explores the relationship between anger and stigmatization. As expected, anger was positively related to both of the indicators for the stigmatization of people infected with COVID-19. People who reported higher anger were more likely to attribute greater responsibility to those infected with COVID-19 (*β* = 0.105 [95% CI = 0.028 to 0.182], *p* = 0.008) and exhibited a more stigmatizing attitude toward them (*β* = 0.129 [95% CI = 0.049 to 0.210], *p* = 0.002). Therefore, H3 was supported. Additionally, none of the other emotions were related to the indicators of stigmatization.

The final hypothesis concerns the relationship between depression and compliance with the government’s social-distancing policy. The analysis revealed somewhat contradictory results. The expected negative relationship was found for support for the “routine-life-distancing” policy (*β* = −0.127 [95% CI = −0.201 to −0.052], *p* = 0.001) and stronger social-distancing policies (*β* = −0.096 [95% CI = −0.173 to −0.018], *p* = 0.015). However, unexpectedly, depression was positively related to the voluntary practice of social distancing (*β* = 0.185 [95% CI = 0.103 to 0.268, *p* = 0.000).

Additionally, among the control variables, support for President Moon was related consistently and significantly to all the dependent variables, suggesting that individuals’ political positions play an important role in policy compliance and stigmatization. These results are summarized in Table 3.

## 4. Discussion

This study explored the relationships between the three most commonly expressed emotional responses (fear, anger, and depression) and two outcome variables (policy compliance and stigmatization) in the context of the COVID-19 pandemic.

This section summarizes the key findings. First, this study found that each emotional response accompanies different behavioral and perceptual consequences, overall consistent with the ATF. Consistent with previous research [11], fear was positively related to all of the indicators of compliance with social-distancing policies. That is, fearful individuals practiced social distancing to a greater extent and supported the government’s current social-distancing policy more so than the other participants. They were even willing to comply with stricter social-distancing rules. Fear appears to serve as an important motivator for protective and preventive behaviors during a pandemic. This result is likely because people were fearful of appraising highly uncertain situations and felt a lack of situational control during the early stage of COVID-19, as the ATF suggests [5].

Regarding anger, the findings suggest that anger does not necessarily inhibit compliance with social distancing, as it was not related to support for either current or future social-distancing policies. Although anger exhibited a negative relationship with the voluntary practice of social distancing, given the nonsignificant results involving the other two compliance indicators, the relationship remains inconclusive. Arguably, this finding could be attributed to a cultural factor: South Korea is considered a collectivistic culture in which compliance is considered a virtue [53]; thus, even though citizens become angry, they may not necessarily resist government policies. Instead, the results suggest that anger may serve as an initiator of social conflicts during a pandemic because it was consistently related to both of the indicators of stigmatization (responsibility attribution and stigmatizing attitude toward people infected with COVID-19). This finding is consistent with previous ATF studies suggesting that anger leads to greater attribution to others [5] and an increase in aggressive behaviors [30]. This phenomenon implies that anger needs to be monitored carefully during a pandemic because it can cause stigmatization, and stigma functions as a major barrier to pandemic control [6].

Regarding depression, the results suggest an interesting contradiction. Depression was negatively related to support for the current social-distancing policies and for stricter social-distancing policies, which is consistent with previous AFT and depression studies. However, it was positively related to the participants’ current social-distancing behaviors. While this finding appears contradictory, the positive relationship between depression and social-distancing practices may be manifesting inverse causality. That is, it may be that those who complied with the government’s policy and actively participated in social distancing experienced greater depression because their freedom had been limited and they became isolated from others. In fact, Wright et al.’s longitudinal study reported a positive relationship between self-reported compliance and depressive symptoms in the middle of COVID-19 among UK adults [54]. When the situation lasted longer than they expected, the participants felt hopeless, and they could have become less supportive of the current social-distancing policy and of any future, stricter social-distancing policy, even though they were complying with the current policy. Therefore, our findings may suggest that dealing with depression is particularly important when the pandemic lasts for a long time. Even among those who have complied with social-distancing policies, their willingness to comply may decrease over time if they experience depression.

The present study provides practical implications for pandemic control. Specifically, this study suggests that it is important to maintain some level of fear among the public to obtain greater compliance with health authorities’ guidelines. Health authorities should be cautious because people can have an optimistic bias; therefore, health authorities should consistently emphasize the threat of the target diseases. Of course, efficacy messages should be delivered together with fear messages, as fear appeal messages tend to be more effective when coupled with self- or response-efficacy components [11].

The findings also imply that anger must be countered to minimize the negative effects of stigmatization and social conflicts. Although stigma has some positive aspects, as it can help people avoid the stigmatized condition, the negative aspect of stigma from the perspective of pandemic control should not be ignored. The literature has suggested several predictors of anger, such as perceptions of responsibility [18,55], fairness [56], and risk [57]. Health authorities need to consider these factors when communicating with the public to ease their anger.

Additionally, the level of depression should be carefully monitored when the pandemic is long lasting. In particular, health authorities need to pay attention to those who have been compliant because their willingness to comply may decrease at certain points if they begin to feel hopelessness. Previous studies suggest that the sense that the future is hopeless is a key factor in the development of depression [58]. In a recent study conducted in the context of the COVID-19 outbreak, hope was positively related to well-being [59]. In this sense, incorporating a message component that delivers hope might be beneficial for communicating risk during a pandemic. Regular updates about what specific measures are being implemented and planned despite the severe situation may deliver, to some extent, a sense of hope to the public. In addition, because the emotional climate in communities can affect individuals’ emotional experiences [60], health authorities need to pay special attention to areas that have been seriously affected or locked down.

This study also presents theoretical implications for future studies. This study suggests the importance of specifying the types of emotional responses in studying crisis or risk communication. Since each discrete emotional response results in different behavioral and perceptual consequences, identifying the relevant types of emotional responses and examining their consequences will allow researchers to provide implications for pandemic control that are more concrete. On the other hand, individuals may experience multiple emotions to varying degrees when encountering a crisis. Therefore, it would be interesting to explore how emotions interact with each other or exert their influence conjointly.

This study has several limitations. First, because we rely on a cross-sectional survey, causality cannot be determined. While we postulated that the positive relationship between depression and current participation in social distancing may suggest inverse causality, such a postulation cannot be supported empirically with the current data. A longitudinal study that examines the changes in depression and policy compliance over time would be able to provide clearer conclusions. Second, some aspects of this study may limit the generalizability of the findings: this study relied on an online survey; thus, the participants may not be representative of the actual population; as emotions are considered a cultural phenomenon [61], the findings in this study may not be generalizable to other countries; a couple of measures (compliance with social-distancing policies and stigma perception) are devised by the authors as standardized measures are not available. Finally, although this study explains the relationships between emotions and their behavioral and perceptual consequences, it provides limited explanations concerning the factors that influence those emotions. In particular, the message factors that arouse fear (such as severity and susceptibility) have been well studied, but little is known about which message components can trigger anger or hope in the pandemic context. Exploring such factors would provide important insights to develop more effective risk communications during the pandemic.

## 5. Conclusions

Throughout the COVID-19 pandemic, health authorities have faced various emotional responses by the public, which have complicated the health authorities’ epidemic control efforts. This study suggests that, for epidemic control, it is important to scrutinize specific types of emotional responses and address with them considering the associated behavioral and perceptual consequences. As public health experts warn of the next potential pandemic, the lessons learned from COVID-19 as implied in this study should be the basis for planning effective risk-communication strategies.

## Figures and Tables

**Table 1:** Demographic Characteristics (*n* = 1000).

Gender	females *n* = 506 (50.6%), males *n* = 494 (49.4%)
Age	(range = 19 to 88) 20 s or below, *n* = 183 (18.3%) 30 s, *n* = 160 (16.0%) 40 s, *n* = 190 (19.0%) 50 s, *n* = 197 (19.7%) 60 s, *n* = 267 (26.7%) 70 s or above, *n* = 3 (.3%)
Educational level	middle school degree or below, *n* = 10 (1.0%) high school degree, *n* = 249 (24.9%) college degree, *n* = 662 (66.2%) graduate school degree, *n* = 79 (7.9%)
Household Income (Million in South Korean won (KRW))	below 1 M, *n* = 56 (5.6%) 1 M to less than 2 M, *n* = 81 (8.1%) 2 M to less than 3 M, *n* = 144 (14.4%) 3 M to less than 4 M, *n* = 194 (19.4%) 4 M to less than 5 M, *n* = 188 (18.8%) 5 M to less than 6 M, *n* = 132 (13.2%) 6 M or above, *n* = 205 (20.5%)
Religion	No religion, *n* = 521 (52.1%) Buddhist, *n* = 170 (17.0%) Catholic, *n* = 120 (12.0%) Christian, *n* = 181 (18.1%) Shincheonji, *n* = 5 (.5%) Other, *n* = 3 (.3%)
Residential area	Seoul, *n* = 191 (19.1%) Gyeonggi, *n* = 309 (30.9%) Chungcheong, *n* = 62 (6.2%) Gangwon, *n* = 31 (3.1%) Jeolla, *n* = 99 (9.1%) Gyeongsang, *n* = 253 (25.3%) Jeju, *n* = 521 (52.1%)

**Table 2:** Descriptive statistics of the key variables (*n* = 1000).

	*M*	*SD*	1	2	3	4	5	6	7	8
Fear	3.35	0.91	(0.90)	0.55 **	0.59 **	0.17 **	0.15 **	0.13 **	0.13 **	0.11 **
2. Anger	3.14	0.98		(0.92)	0.59 **	0.07 *	0.04	0.05	0.12 **	0.15 **
3. Depression	2.63	0.98			(0.90)	0.17 **	−0.05	−0.01	0.04	0.11 **
4. Voluntary practice of social distancing	3.62	0.98				(0.65)	0.33 **	0.30 **	0.15 **	0.16 **
5. Support for the “routine-life-distancing” policy	4.34	0.74					(0.69)	0.48	0.32 **	0.15 **
6. Support for stronger social-distancing policies	4.34	0.72						(0.79)	0.36 **	0.19 **
7. Attribution	4.33	0.70							(0.77)	0.39 **
8. Stigma perception	3.64	0.96								(0.88)

Note. Pearson’s correlation coefficients. * *p* < 0.05, ** *p* < 0.01.

**Table 3:** (**a**) The results of the regression analysis (*n* = 1000) (DV: Compliance with the social-distancing policy); (**b**) The results of the regression analysis (*n* = 1000) (DV: Stigmatization).

(a)			
	*β* [95% Confidence Interval]		
	Voluntary Practice of Social Distancing	Support for the “Routine-Life-Distancing” Policy	Support for Stronger Social-Distancing Policies
Gender	−0.032 [−0.094, 0.030]	0.119 *** [0.063, 0.175]	0.109 *** [0.051, 0.166]
Age	0.108 [0.045, 0.171]	0.120 *** [0.063, 0.117]	0.103 ** [0.044, 0.162]
Education	0.025 [−0.039, 0.089]	0.035 [−0.023, 0.093]	0.003 [−0.057, 0.064]
Income	−0.033 [−0.095, 0.030]	0.019 [−0.038, 0.075]	−0.004 [−0.063, 0.055]
*R*^2^	0.009	0.054 ***	0.033 ***
Religion	−0.030 [−0.092, 0.032]	−0.009 [−0.065, 0.047]	−0.001 [−0.057, 0.057]
Residence	−0.042 [−0.104, 0.019]	0.006 [−0.050, 0.061]	0.041 [−0.017, 0.098]
Confirmed case	−0.053 [−0.116, 0.009]	−0.008 [−0.064, 0.048]	0.029 [−0.029, 0.087]
Support for President Moon	0.076 * [0.015, 0.138]	0.181 *** [0.125, 0.236]	0.312 *** [0.255, 0.369]
Severity	0.096 ** [0.044, 0.31]	0.351 *** [0.292, 0.410]	0.192 *** [0.131, 0.253]
Susceptibility	−0.040 [−0.104, 0.024]	−0.062 * [−0.120, −0.004]	0.012 [−0.048, 0.071]
*ΔR*^2^	0.026 ***	0.179 ***	0.149 ***
Depression	0.185 *** [0.103, 0.268]	−0.127 ** [−0.201, −0.052]	−0.096 * [−0.173, −0.018]
Anger	−0.083 * [−0.162, −0.004]	0.027 [−0.044, 0.099]	0.053 [−0.021, 0.127]
Fear	0.095 * [0.011, 0.179]	0.114 ** [0.038, 0.189]	0.088 * [0.010, 0.167]
*ΔR*^2^	0.039 ***	0.011 **	0.008 *
*Total R^2^ (R^2^_adj_)*	0.074 (0.062) ***	0.244 (0.234) ***	0.190 (0.179) ***
(b)			
	*β* [95% Confidence Interval]		
	Attribution	Stigma Perception	
Gender	0.001 [−0.059, 0.061]	−0.039 [−0.102, 0.024]	
Age	−0.002 [−0.063, 0.060]	0.037 [−0.027, 0.102]	
Education	0.005 [−0.058, 0.067]	−0.042 [−0.108, 0.023]	
Income	−0.006 [−0.067, 0.055]	−0.039 [−0.103, 0.025]	
*R*^2^	0.002	0.004	
Religion	0.026 [−0.034, 0.087]	−0.001 [−0.064, 0.062]	
Residence	0.023 [−0.037, 0.082]	−0.010 [−0.072, 0.053]	
Confirmed case	−0.004 [−0.064, 0.057]	0.018 [−0.046, 0.081]	
Support for President Moon	0.225 *** [0.165, 0.284]	0.080 * [0.018, 0.143]	
Severity	0.224 *** [0.161, 0.288]	0.040 [−0.026, 0.107]	
Susceptibility	−0.027 [−0.089, 0.036]	−0.007 [−0.072, 0.058]	
*ΔR*^2^	0.112 ***	0.011	
Depression	0.041 [−0.121, 0.040]	0.043 [−0.041, 0.127]	
Anger	0.105 ** [0.028, 0.182]	0.129 ** [0.049, 0.210]	
Fear	−0.041 [−0.041, 0.122]	0.009 [−0.076, 0.094]	
*ΔR*^2^	0.010 **	0.023 ***	
*Total R^2^ (R^2^_adj_)*	0.124 (0.112) ***	0.038 (0.025) ***	

Note. All the coefficients are standardized. * *p* < 0.05, ** *p* < 0.01, *** *p* < 0.001.

## Data Availability

Qualified researchers can obtain the data from the corresponding author (jhchoi@hallym.ac.kr). The data are not publicly available due to privacy concerns imposed by the IRB.
